# Environmental Factors That Affect Parathyroid Hormone and Calcitonin Levels

**DOI:** 10.3390/ijms23010044

**Published:** 2021-12-21

**Authors:** Mirjana Babić Leko, Nikolina Pleić, Ivana Gunjača, Tatijana Zemunik

**Affiliations:** Department of Medical Biology, School of Medicine, University of Split, Šoltanska 2, 21000 Split, Croatia; mbabic@mefst.hr (M.B.L.); npleic@mefst.hr (N.P.); igunjaca@mefst.hr (I.G.)

**Keywords:** environmental factors, PTH, calcitonin, pollutants, lifestyle factors, calcium, phosphate, vitamin D

## Abstract

Calciotropic hormones, parathyroid hormone (PTH) and calcitonin are involved in the regulation of bone mineral metabolism and maintenance of calcium and phosphate homeostasis in the body. Therefore, an understanding of environmental and genetic factors influencing PTH and calcitonin levels is crucial. Genetic factors are estimated to account for 60% of variations in PTH levels, while the genetic background of interindividual calcitonin variations has not yet been studied. In this review, we analyzed the literature discussing the influence of environmental factors (lifestyle factors and pollutants) on PTH and calcitonin levels. Among lifestyle factors, smoking, body mass index (BMI), diet, alcohol, and exercise were analyzed; among pollutants, heavy metals and chemicals were analyzed. Lifestyle factors that showed the clearest association with PTH levels were smoking, BMI, exercise, and micronutrients taken from the diet (vitamin D and calcium). Smoking, vitamin D, and calcium intake led to a decrease in PTH levels, while higher BMI and exercise led to an increase in PTH levels. In terms of pollutants, exposure to cadmium led to a decrease in PTH levels, while exposure to lead increased PTH levels. Several studies have investigated the effect of chemicals on PTH levels in humans. Compared to PTH studies, a smaller number of studies analyzed the influence of environmental factors on calcitonin levels, which gives great variability in results. Only a few studies have analyzed the influence of pollutants on calcitonin levels in humans. The lifestyle factor with the clearest relationship with calcitonin was smoking (smokers had increased calcitonin levels). Given the importance of PTH and calcitonin in maintaining calcium and phosphate homeostasis and bone mineral metabolism, additional studies on the influence of environmental factors that could affect PTH and calcitonin levels are crucial.

## 1. Introduction

**Figure 1:**
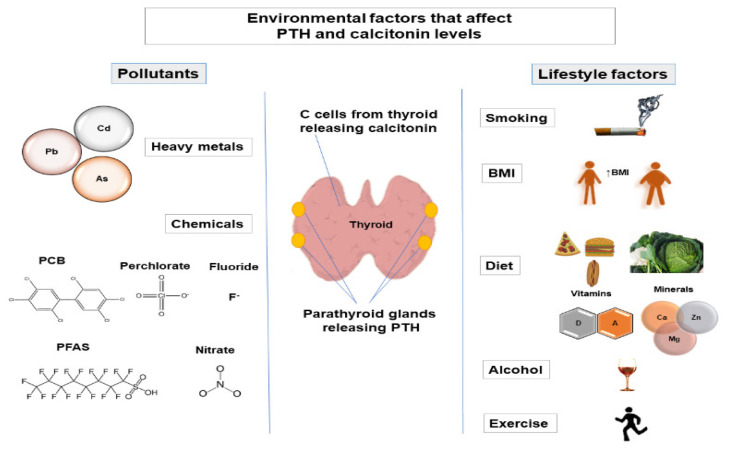
Environmental factors (lifestyle factors and pollutants) that affect PTH and calcitonin levels. As, arsenic; BMI, body mass index; Ca, calcium; Cd, cadmium; F, fluoride; Mg, magnesium; Pb, lead; PCB, polychlorinated biphenyl; PFAS, perfluoroalkyl substances; PTH, parathyroid hormone; Zn, zinc.

Maintenance of calcium homeostasis in the body is crucial since calcium regulates various physiological processes, including cellular signaling, protein and enzyme function, neurotransmission, contractility of the muscles, and blood coagulation [1]. Calcium homeostasis is regulated by parathyroid hormone (PTH), calcitonin, the active form of vitamin D (1α,25-dihydroxyvitamin D (1,25(OH)2D3)), and serum calcium and phosphate levels. Regulation of phosphate metabolism is also important as phosphate is involved in protein and enzyme function, cell signaling, and skeletal mineralization and is a component of cell membranes and nucleic acids [2,3]. The main factors that regulate phosphate homeostasis are PTH, fibroblast growth factor 23 (FGF-23), 1,25(OH)2D3, and Klotho [3]. Calcitonin is also involved in the regulation of phosphate levels [4,5]. PTH is released from the parathyroid glands [6], while calcitonin is released from thyroid C-cells [7]. Alternation of PTH levels can lead to the development of hyperparathyroidism and hypoparathyroidism. Changes in calcitonin levels have also been observed in pathological conditions (such as medullary thyroid carcinoma [8]). Therefore, variations in PTH and calcitonin levels may indicate that the normal functioning of parathyroid glands and thyroid is altered. Various factors can affect PTH and calcitonin levels, such as genetic factors [9,10,11], demographic factors (age [12,13,14], sex [15,16,17]), and environmental factors [18,19,20,21]. It is estimated that genetic factors account for 60% of variations in PTH levels [9], while the amount to which genetic factors contribute to interindividual variation in calcitonin levels has not been studied. This review aims to provide an insight into environmental factors (lifestyle factors and pollutants) that affect PTH and calcitonin levels (Figure 1).

## 2. Involvement of PTH and Calcitonin in the Regulation of Calcium and Phosphate Levels

Calcium and phosphate levels in the body are regulated by the complex intestine–bone–kidney–parathyroid axis [22]. Calcium homeostasis is regulated by PTH, calcitonin, 1,25(OH)2D3, and serum phosphate and calcium levels. PTH increases calcium levels in the body, and calcitonin decreases calcium levels in the body. PTH increases serum calcium levels by activating osteoclasts (cells involved in bone resorption) and absorbing calcium in the kidneys. Calcitonin lowers calcium levels by inhibiting osteoclasts [23]. Additionally, 1,25(OH)2D3 stimulates intestinal calcium absorption [24]. Increasing serum levels of 1,25(OH)2D3 and calcium decrease PTH secretion, while increasing serum phosphate levels increase PTH secretion [25]. In addition to PTH, phosphate levels are mainly regulated by FGF-23, 1,25(OH)2D3, Klotho, and dietary phosphate [3,22,26,27], while calcitonin also affects phosphate levels [4,5]. PTH, FGF-23, and Klotho decrease serum phosphate levels (by inhibiting renal phosphate reabsorption), while 1,25(OH)2D3 increases serum phosphate levels (by increasing renal phosphate reabsorption, phosphate absorption from the intestine, and phosphate release from the bones) [2,22]. It has been suggested that FGF-23 acts in a negative feedback loop with PTH [28]; PTH stimulates FGF-23 production [28], while FGF-23 has been shown to inhibit PTH secretion indirectly (by increasing urinary phosphate excretion) and directly (by acting directly on parathyroid glands) [29]. Additionally, a negative feedback mechanism was observed between FGF-23 and 1,25(OH)2D3; 1,25(OH)2D3 increases FGF-23 levels, and FGF-23 decreases 1,25(OH)2D3 levels (by suppressing the expression of 1α-hydroxylase—the enzyme responsible for the production of 1,25(OH)2D3) (reviewed in [22]).

## 3. Environmental Factors That Affect PTH and Calcitonin Levels

### 3.1. Lifestyle Factors

#### 3.1.1. Smoking

Many studies have investigated the impact of smoking on PTH levels. Most of these studies reported a decrease in PTH levels in smokers (Table 1). The three largest studies that involved more than 7000 participants confirmed these results [30,31,32]. The study of Diaz-Gomez et al., even showed that maternal smoking decreases PTH levels in newborns [33]. The heavy metal cadmium and thiocyanate (that is converted from cyanide in tobacco) which are also toxic components of tobacco smoke have been shown to reduce PTH levels [19,34]. Jorde et al., observed that after smoking cessation, PTH levels return to normal [30]. The mechanism by which smoking affects PTH levels is not fully understood. PTH–vitamin D axis dysfunction has been observed in smokers [35]. Many studies have found a decrease in 1,25(OH)2D levels among smokers (reviewed in [36]). Although under physiological conditions, a decrease in 1,25(OH)2D levels was accompanied by an increase in PTH levels, this was not observed in smokers in most studies. Need et al., suggested that smoking impairs osteoblast function, increasing serum calcium, which in turn leads to a decrease in PTH levels [37]. Jorde et al. did not rule out a possible direct toxic effect of smoking on parathyroid cells [30]. Additionally, it has been suggested that a decrease in bone mineral density (BMD) among smokers [38] may contribute to PTH–vitamin D axis dysfunction [35].

Most studies investigating the effect of smoking on calcitonin levels have found an increase in calcitonin levels in smokers (Table 2). A large population study by Song et al., involving 10,566 participants showed an increase in calcitonin levels in male smokers [17]. Smoking affects the normal functioning of the thyroid gland [39]; however, the effect of smoking on calcitonin-producing C cells has not been elucidated [17]. The results of Tabassian et al. suggested that the lungs are the source of increased calcitonin in smokers rather than the thyroid. Specifically, smoking increases the release of calcitonin from neuroendocrine lung cells [40].

#### 3.1.2. Body Mass Index

Many studies have investigated the influence of body mass index (BMI) on PTH levels. Most studies have shown that an increase in BMI is accompanied by an increase in PTH levels (Table 1). However, a study by Yuan et al., showed a positive correlation between BMI and PTH levels in subjects with lower PTH levels (below 65.8 pg/mL), while a negative correlation was observed between BMI and PTH levels in the group of patients with high PTH levels (above 147 pg/mL) [41]. There are several possible explanations for the positive correlation between BMI and PTH levels. The first possibility is that weight gain leads to an increase in PTH levels by sequestration of 25-hydroxyvitamin D (25(OH)D) in adipose tissue (since 25(OH)D is soluble in fat) [42,43]. Because PTH and 25(OH)D are inversely related, a decrease in 25(OH)D levels increases PTH levels. Another possibility is that an increase in PTH levels causes weight gain. Because PTH can activate 1α-hydroxylase (the enzyme responsible for the production of 1,25(OH)2D), an increase in PTH levels can lead to an increase in 1,25(OH)2D levels. Both PTH and 1,25(OH)2D increase calcium levels. Increased calcium levels in adipocytes result in increased lipid storage (by activation of phosphodiesterase 3β which reduces catecholamine-induced lipolysis [44,45]). A possible explanation of the negative correlation between PTH and BMI in patients with high PTH levels is that PTH in higher concentrations inhibits adipogenesis, consequently resulting in weight loss [46]. Additionally, high-dose PTH has been shown to increase the expression of thermogenesis genes, resulting in white adipose browning [47].

Several studies have investigated the association between BMI and calcitonin levels, reporting conflicting results (Table 2). The largest study, which included 9340 people with type 2 diabetes, showed a positive correlation between BMI and calcitonin levels [48]. However, a study by Song et al., conducted on 4638 healthy individuals did not show an association between BMI and calcitonin [17]. Although the relationship between calcitonin levels and BMI in humans has not been fully elucidated, experimental studies have shown that salmon calcitonin intake causes weight loss (reviewed in [49]). These authors also described some additional compounds that target the calcitonin receptor and that could be used as an option in the treatment of obesity [49].

#### 3.1.3. Diet

Different types of food can affect the level of PTH in the body (Table 1). A diet high in phosphorus and low in calcium has been shown to increase PTH levels [50,51]. This is logical because both high serum phosphate levels and low serum calcium levels are signals to increase PTH release [52]. Phosphorus is present in various types of food and food additives, while dairy products contain a large amount of calcium. Increased intake of dairy products and decreased intake of highly processed food should increase calcium levels and reduce phosphorus levels [51]. Processed foods such as sausages, salami, and white bread [21] and a proinflammatory diet (processed and red meat, refined carbohydrates, and fried food) [53] have been observed to increase PTH levels. Consumption of this type of food increases BMI, which is positively correlated with PTH levels (Table 1). A decrease in PTH levels was observed in consumers of bran bread [21]. A low–protein diet was associated with an increase in PTH levels [54,55,56]. Interestingly, the consumption of plant foods also led to an increase in PTH levels [21,57]. Therefore, vegans [58] and vegetarians [59] had higher levels of PTH than controls. A possible explanation for this is that higher plant food intake increases serum phosphorus levels (due to pesticide treatment of plants) [60]. PTH levels either decreased [30,61] or did not change [32,62,63] after coffee consumption.

The effect of different types of food on calcitonin levels has not been studied to date. Several studies have shown that food intake (without specifying the type of food) does not affect calcitonin levels [64,65]. Zayed et al., have shown that calcitonin levels increase after ingestion of food (without specifying the type of food) [66]. A study in pigs showed that a diet high in phosphorus increased calcitonin levels [67], while a study in rats showed that a diet high in fat increased calcitonin levels [68].

##### Micronutrients

Many studies have tested the effect of vitamin D on PTH levels because these two hormones act together. About 95% of vitamin D is synthesized in the skin after exposure to sunlight, while 5% of vitamin D comes from food [69]. Since PTH and the active form of vitamin D (1,25(OH)2D) are in an inverse relationship, it is not surprising that most of the studies have reported a decrease in PTH levels after vitamin D intake (Table 1). In some studies, however, there was no change in PTH levels after vitamin D intake (Table 1). On the other hand, a meta-analysis by Moslehi et al. confirmed that PTH levels are reduced by vitamin D intake [70]. Vitamin A intake decreased [63,71] or did not affect PTH levels [72]. In vitro studies in human [73] and bovine parathyroid cells [74] have shown that retinoic acid (a metabolite of vitamin A) directly suppresses PTH secretion.

No changes in calcitonin levels were observed after vitamin D intake [75]. While calcitonin stimulates 1,25(OH)2D synthesis, 1,25(OH)2D reduces the synthesis of calcitonin [76]. Therefore, it is necessary to conduct additional studies on the relationship between vitamin D and calcitonin.

Most studies have shown that calcium intake decreases PTH levels (Table 1), which is logical since PTH is released in hypocalcemia. Magnesium intake either increased [77,78] or did not affect [32,79] PTH levels. The relationship between PTH and magnesium is complex because PTH improves magnesium absorption [80], and magnesium reduces PTH secretion in a state of moderately low calcium concentration [81,82]. Zinc intake [83] did not affect PTH levels. However, a study in rats showed that a zinc-deficient diet increased PTH levels [84], while patients with primary hyperparathyroidism had decreased serum zinc levels [85].

Zinc intake decreased calcitonin levels [83,86], while copper intake [86] did not affect calcitonin levels. Intake of both zinc and copper resulted in inhibition of bone loss [87,88].

#### 3.1.4. Alcohol

Studies investigating the influence of alcohol on PTH levels have yielded conflicting results. Some studies have found a decrease in PTH levels in alcoholics, while most studies have not reported a significant change in PTH levels due to alcohol consumption (Table 1). Moreover, the two largest studies involving more than 7000 participants yielded conflicting results; Jorde et al. observed a significant reduction in PTH levels in alcoholics [30], while Paik et al. did not notice a significant change in PTH levels in alcoholics [32]. Because alcohol inhibits bone regeneration [89], it has been suggested that alcohol intake reduces PTH levels [90,91,92] and increases calcitonin levels [93].

Several studies investigated calcitonin levels in alcoholics, and all yielded conflicting results (Table 2) with calcitonin levels that were increased [94], decreased [95], or unchanged [96] in alcoholics. Schuster et al. suggested that the reduction in calcitonin in chronic alcoholism is due to lower calcium concentration at this stage of alcohol consumption [95]. Interestingly, animal studies have shown that salmon calcitonin intake reduces various alcohol-related behaviors [97,98].

#### 3.1.5. Exercise

Most studies that have investigated the influence of exercise on PTH levels have reported an increase in PTH levels during and after exercise (Table 1). However, most of these studies involved a small number of participants (less than 50). In contrast to the results of these studies, two studies involving as many as 7561 [31] and 3427 [30] participants reported a decrease in PTH levels after exercise. Causes of inconsistencies between studies may be the physical status of the participants; the age and gender of the participants; and the type, duration, and intensity of the exercise [99]. PTH is thought to increase during high-intensity exercise (reviewed in [100]). Although exercise is thought to be beneficial for BMD, some groups of professional athletes have had significant reductions in BMD [101,102]. It has been suggested that intense exercise leads to a decrease in calcium levels, resulting in an increase in PTH. Elevated PTH levels may contribute to bone resorption (reviewed in [103]). Moreover, Shea et al. suggested that calcium supplementation during exercise could reduce bone resorption [104]. However, other researchers have noticed an increase in PTH levels during exercise despite the stability of calcium levels (reviewed in [103]). Some other factors that can lead to an increase in PTH during exercise are increased catecholamine release (which stimulates PTH release) [105], increased aldosterone release (which increases PTH and calcitonin release) [80], and acidosis (stimulates PTH release) [106].

Calcitonin levels increased [107,108] or did not change [20,109,110,111,112] during exercise. However, these results should be verified in larger cohorts as most of these studies involved less than 30 participants (Table 2). Calcitonin levels could increase during exercise due to an increase in aldosterone levels [80].

**Table 1:** Lifestyle factors that affect PTH levels in humans.

	Factor	Effect on Hormone Levels	Number of Participants	Participants	Reference
**Smoking**	Smoking	↓PTH	170 (men)	Healthy adults	[113]
	Smoking	↓PTH	376	Healthy adults	[114]
	Smoking	↓PTH	510	Healthy adults	[62]
	Smoking	↔PTH	535	Healthy adults	[115]
	Smoking	↔PTH	1203	Healthy adults	[116]
	Smoking	↓iPTH	177	Healthy adults	[117]
	Smoking	↓PTH (in mothers and their new-borns)	61	Mothers and their new-borns	[33]
	Smoking	↓iPTH	31 (men)	Healthy adults	[118]
	Smoking	↔iPTH	43 (women)	Healthy adults	[118]
	Smoking	↓PTH	7896	Healthy adults	[30]
	Smoking	↓PTH	405 (women)	Healthy adults	[37]
	Smoking	↓PTH	958 (men)	Healthy adults	[119]
	Smoking	↔PTH	136	Healthy adults	[92]
	Smoking	↓PTH	406	Healthy adults	[38]
	Smoking	↔PTH	3212	2758 healthy adults + 454 participants with coronary heart disease	[120]
	Smoking	↓iPTH	347	Healthy adults	[61]
	Smoking	↔PTH	1206	Healthy adults	[121]
	Smoking	↔PTH	1068	Healthy adults	[122]
	Smoking	↓iPTH	345	216 healthy adults + 129 men with earlier partial gastrectomy	[123]
	Smoking	↓PTH	7561	Healthy adults	[31]
	Smoking	↓iPTH	3949	Healthy adults	[124]
	Smoking	↔PTH	32	Healthy adults	[125]
	Smoking	↓PTH	1288	Healthy adults	[63]
	Smoking	↓PTH	7652	Healthy adults	[32]
	Smoking	↔PTH	414	Healthy adults	[126]
	Smoking	↓PTH	2810	Healthy adults	[127]
	Smoking	↔PTH	1205	Healthy adults	[128]
		↔PTH	719 (men)		
	Smoking	↑PTH	128 (participants with low body weight (≤75 kg))	Healthy adults	[129]
	Smoking	↓PTH	1067 (women)	Healthy adults	[130]
	Smoking	↓PTH	47 (women)	Healthy adults	[131]
	Smoking	↔PTH	489 (women)	Healthy adults	[91]
	Smoking	↓PTH	908	Healthy adults	[132]
	Smoking	↓PTH	294 (women)	Healthy adults	[18]
	Smoking	↔PTH	58	Healthy adults	[133]
**Alcohol consumption**	Alcohol	↔PTH	535	Healthy adults	[115]
	Alcohol	↔PTH	510	Healthy adults	[62]
	Alcohol	↔PTH	1203	Healthy adults	[116]
	Alcohol	↓PTH	7896	Healthy adults	[30]
	Alcohol	↓PTH	136	Healthy adults	[92]
	Alcohol	↔PTH	1206	Healthy adults	[121]
	Alcohol	↓iPTH	3949	Healthy adults	[124]
	Alcohol	↔PTH	1288	Healthy adults	[63]
	Alcohol	↔PTH	414	Healthy adults	[126]
	Alcohol	↔PTH	1205	Healthy adults	[128]
	Alcohol	↔PTH	7652	Healthy adults	[32]
	Alcohol	↔PTH	27 (men)	Healthy adults, alcoholics	[134]
	Alcohol	↔PTH	21 (men)	Healthy adults, alcoholics	[135]
	Alcohol	↓PTH	6	Healthy adults	[90]
	Alcohol	↔PTH	47	Healthy adults, alcoholics	[95]
	Alcohol	↔PTH	26	Healthy adults	[136]
	Alcohol	↓PTH	136	Healthy adults	[92]
	Alcohol	↓PTH (increase in PTH levels after alcohol withdrawal)	26	Healthy adults, alcoholics	[137]
	Alcohol	↔iPTH	36 (men)	Healthy adults, alcoholics	[138]
	Alcohol	↓immunoreactive PTH	104 (men)	Healthy adults	[139]
**Increased BMI**	↑BMI	↔PTH	535	Healthy adults	[115]
	↑BMI	↑PTH	510	Healthy adults	[62]
	↑BMI	↑PTH	1203	Healthy adults	[116]
	↑BMI	↑PTH	7896	Healthy adults	[30]
	↑BMI	↑PTH	7561	Healthy adults	[31]
	↑BMI	↑PTH	3212	2758 healthy adults + 454 participants with coronary heart disease	[120]
	↑BMI	↑iPTH	347	Healthy adults	[61]
	↑BMI	↑PTH	1206	Healthy adults	[121]
	↑BMI	↑PTH	2810	Healthy adults	[127]
	↑BMI	↑PTH	1205	Healthy adults	[128]
	↑BMI	↑PTH	7652	Healthy adults	[32]
	↑BMI	↑PTH	1288	Healthy adults	[63]
	↑BMI	↑iPTH	3949	Healthy adults	[124]
	↑BMI	↑iPTH	160	Healthy adults	[140]
	↑BMI	↑PTH	483	Healthy adults	[141]
	↑BMI	↔PTH	57	Healthy adults	[79]
	↑BMI	↑PTH	57 (men)	Healthy adults	[142]
	↑BMI	↑PTH	1628	Dialysis patients	[143]
	↑BMI	↑PTH	419	Children	[144]
	↑BMI	↑PTH	82 (women)	Healthy adults	[145]
	↑BMI	↑PTH	316	Healthy adults	[146]
	↑BMI	↑iPTH	332	Healthy adults	[147]
	↑BMI	↑PTH	40	Bariatric surgery patients and healthy controls	[148]
	↑BMI	↑PTH	316	Patients who had attended the obesity clinics	[149]
	↑BMI	↑PTH	42	Patients undergoing sleeve gastrectomy	[150]
	↑BMI	↑PTH	516	Healthy adults	[151]
	↑BMI	↑PTH	3248 (women)	Healthy adults	[152]
	↑BMI	↑PTH	669 (men)	Healthy adults	[153]
	↑BMI	↑iPTH	590	Hemodialysis patients	[154]
	↑BMI	↑PTH	2758 healthy adults + 454 participants with coronary heart disease	Healthy adults	[155]
	↑BMI	↑PTH	250	Healthy adults	[156]
	↑BMI	↑PTH	608	Healthy adults	[157]
	↑BMI	↑PTH	496 (men)	Patients with chronic kidney disease	[158]
	↑BMI	↔PTH	1436	Healthy adults	[159]
	↑BMI	↑PTH	304 (women)	Healthy adults	[160]
	↑BMI	↑PTH	156	Obese children	[161]
	↑BMI	↑PTH	3002	Healthy adults	[162]
	↑BMI	↑PTH	810 (women)	Healthy adults	[163]
	↑BMI	↑PTH (PTH = 21.4–65.8 pg/ mL)	131	Healthy adults and subjects with primary hyperparathyroidism	[41]
		↓PTH (PTH = 147–2511.7 pg/mL)	132		
	↑BMI	↑PTH	383 (women)	Healthy adults	[164]
	↑BMI	↑PTH	2848	Healthy adults	[165]
	↑BMI	↑PTH	453	Healthy adults	[166]
	↑BMI	↑PTH	25	Anorexia nervosa patients	[167]
	↑BMI	↑PTH	98	Healthy adults	[168]
	↑BMI	↑PTH	625	Healthy adults	[71]
	↑BMI	↑PTH	294	Healthy adults	[18]
**Diet**	Different sorts of vegetables, sausages, salami, mushrooms, eggs, white bread	↑PTH	1180	Healthy adults	[21]
	Bran bread	↓PTH			
	Traditional Inuit diet (diet mainly of marine origin taken by Greenland inhabitants)	↓PTH	535	Healthy adults	[115]
	↑Total calorie intake	↔iPTH	3949	Healthy adults	[124]
	Protein intake	↔PTH	7652	Healthy adults	[32]
	Coronary Health Improvement Project (CHIP). CHIP intervention, which promotes a plant-based diet with little dairy intake and meat consumption	↑PTH (after 6 weeks)	119 (women)	Healthy adults	[57]
	High-phosphorus, low-calcium diets	↑PTH	16	Healthy adults	[50]
	The traditional Brazilian diet (fruits, vegetables, and small amounts of meat)	↓PTH	111	Severely obese adults	[169]
	Extra virgin olive oil supplementation	↔PTH	111	Severely obese adults	[169]
	Moderate dietary protein restriction	↑PTH	18	Patients with idiopathic hypercalciuria and calcium nephrolithiasis	[55]
	Vegans vs omnivores	↑PTH in vegans	155	Healthy adults	[58]
	The “Dietary Approaches to Stop Hypertension” (DASH) diet, rich in fiber and low-fat dairy	↔PTH	334	Healthy adults	[170]
	Vegans vs. omnivores	↔PTH	210 (women)	Healthy adults	[171]
	High protein and high dairy group	↓PTH	30 (women)	Healthy adults	[56]
	Adequate protein and medium dairy group	↓PTH	30 (women)	Healthy adults	[56]
	Adequate protein and low dairy	↑PTH	30 (women)	Healthy adults	[56]
	Diet with low calcium:phosphorus ratio	↑PTH	147 (women)	Healthy adults	[51]
	Low-protein diets (diets containing 0.7 and 0.8 g protein/kg)	↑PTH	8 (women)	Healthy adults	[54]
	Higher consumption of a proinflammatory diet	↑PTH	7679	Adults with/without chronic kidney disease	[53]
	High fruit and vegetable intake (consuming more than 3 servings of fruit and vegetables)	↓PTH	56	Children	[172]
	Dietary calorie, vitamin D, and magnesium intake	↔PTH	98	Healthy adults	[168]
	Vegetarians vs. controls	↑iPTH	44	Healthy adults	[59]
	Intake of dietary fiber	↑iPTH			
	Dietary calcium intake	↓iPTH			
	Coffee	↓iPTH	181 (men)	Healthy adults	[61]
	Coffee, tea	↔PTH	510	Healthy adults	[62]
	Coffee	↓PTH	3427 (men)	Healthy adults	[30]
	Caffeine intake	↔PTH	7652	Healthy adults	[32]
	Caffeine intake	↔PTH	1288	Healthy adults	[63]
	Vitamin D supplements	↔PTH	510	Healthy adults	[62]
	Vitamin D supplements	↓PTH	4469 (women)	Healthy adults	[30]
	Vitamin D supplements	↓iPTH	3949	Healthy adults	[124]
	Vitamin D supplements	↔PTH	1288	Healthy adults	[63]
	Vitamin D supplements	↓PTH	414	Healthy adults	[126]
	Vitamin D intake	↓PTH	316	Healthy adults	[146]
	Vitamin D supplementation	↓PTH	250	Healthy adults	[156]
	Vitamin D intake	↓PTH	376 (women)	Healthy adults	[173]
	Vitamin D supplementation	↓PTH	Meta-analysis		[70]
	Vitamin D and calcium supplementation	↓PTH	77	Healthy adults	[174]
	Vitamin D and calcium supplementation	↓PTH	247 (women)	Healthy adults	[175]
	Vitamin D and calcium supplementation	↓PTH	877 (women)	Healthy adults	[176]
	Vitamin D supplementation	↓PTH	270 (women)	Healthy adults	[75]
	Vitamin D and calcium supplementation	↓PTH	313	Healthy adults	[177]
	Vitamin D and calcium supplementation	↓PTH	103 (women)	Elderly institutionalised women	[178]
	Vitamin D supplementation	↔PTH	128 (women)	Healthy adults	[179]
	Vitamin D and calcium supplementation	↓PTH	145 (women)	Healthy adults	[180]
	Vitamin D supplementation	↓PTH	60 (men)	Healthy adults	[181]
	Vitamin D and calcium supplementation	↓PTH	192 (women)	Healthy adults	[182]
	Vitamin D and calcium supplementation	↓PTH	191 (women)	Ambulatory elderly women	[183]
	Vitamin D supplementation	↔PTH	208 (women)	Healthy adults	[184]
	Vitamin D and calcium supplementation	↓PTH	314	Healthy adults	[185]
	Vitamin D and calcium supplementation	↓PTH	1368	Healthy adults	[127]
	Vitamin D supplementation	↓PTH	338	Healthy adults	[186]
	Vitamin D and calcium supplementation	↓PTH	218	Older patients	[187]
	Vitamin D supplementation	↔PTH	215	Healthy adults	[188]
	Vitamin D and calcium supplementation	↓PTH	242	Healthy adults	[189]
	Vitamin D supplementation	↓PTH	165	Healthy overweight subjects	[190]
	Vitamin D and calcium supplementation	↓PTH	153	Healthy adults	[191]
	Multiple micronutrient and calcium supplementation	↓PTH	153 (women)	Healthy adults	[191]
	Vitamin D and calcium supplementation	↓PTH	158	Overweight subjects	[192]
	Vitamin D supplementation	↓PTH	202	Healthy adults	[193]
	Vitamin D supplementation	↓PTH	94	Healthy adults	[194]
	Vitamin D supplementation	↔PTH	90	Coronary artery disease patients	[195]
	Vitamin D supplementation	↔PTH	151	Healthy adults	[196]
	Vitamin D supplementation	↓PTH	89	Obese with pre- or early diabetes	[197]
	Vitamin D supplementation	↓PTH	112	Hypertensive patients	[198]
	Vitamin D supplementation	↓PTH	230	Adults with depression	[199]
	Vitamin D supplementation	↓PTH	77 (women)	Healthy adults	[200]
	Vitamin D and calcium supplementation	↓PTH	173 (women)	Healthy adults	[201]
	Vitamin D supplementation	↓PTH	112	Parkinson disease	[202]
	Vitamin D supplementation	↔PTH	82	Healthy adults	[203]
	Vitamin A intake	↔PTH	606	Healthy adults	[72]
	Total calcium and vitamin A intake	↓PTH	625	Healthy adults	[71]
	Vitamin A intake	↓PTH	1288	Healthy adults	[63]
	The dietary intake of minerals (calcium, phosphate, and magnesium) and vitamin D	↔PTH	127	Healthy adults	[204]
	Calcium supplements	↓PTH	414	Healthy adults	[126]
	Calcium supplements	↓PTH	51	Toddlers	[205]
	Calcium intake	↓PTH	7896	Healthy adults	[30]
	Dietary calcium intake	↓PTH	181	Healthy adolescents	[206]
	Calcium intake	↓PTH	1203	Healthy adults	[116]
	Calcium intake	↓PTH	3212	2758 healthy adults + 454 participants with coronary heart disease	[120]
	Calcium intake	↔PTH	1288	Healthy adults	[63]
	Calcium intake	↓iPTH	3949	Healthy adults	[124]
	Dietary calcium intake	↓PTH	7652	Healthy adults	[32]
	Calcium intake	↔PTH	57	Healthy adults	[79]
	Animal/total calcium intake	↓PTH	316	Healthy adults	[146]
	Dietary calcium	↔PTH	155 (women)	Healthy adults	[207]
	Calcium supplements	↓PTH	566	Healthy adults	[208]
	Intake of calcium	↓PTH	82	Healthy adults	[203]
	Calcium intake derived from milk	↓PTH	245 (women)	Healthy adults	[173]
	Magnesium intake	↔PTH	57	Healthy adults	[79]
	Magnesium intake	↔PTH	7652	Healthy adults	[32]
	Magnesium supplementation	↑PTH	10 (patients with hypoparathyroidism)	Patients with osteoporosis	[78]
		↓PTH	10 (patients with vitamin D insufficiency)		
	Magnesium supplementation	↑iPTH	23	Children with diabetes	[77]
	Zinc infusion	↔PTH	38	Patients of short stature, diabetes mellitus, and controls	[83]
	Phosphorus intake	↔PTH	7652	Healthy adults	[32]
	Intervention group (exercise, vitamin D, calcium, protein supplementation)	↓iPTH	220	Patients that were on bariatric surgery	[209]
**Exercise**	Exercise	↓PTH	7561	Healthy adults	[31]
	Exercise	↔PTH	1288	Healthy adults	[63]
	Exercise	↓PTH	3427 (men)	Healthy adults	[30]
	Exercise	↔PTH	414	Healthy adults	[126]
	Exercise	↔PTH	1205	Healthy adults	[128]
	↑Sitting	↑PTH	566	Healthy adults	[208]
	Exercise	↓PTH	625	Healthy adults	[71]
	Exercise	↑PTH	12 (men)	Healthy adults	[210]
	Exercise	↑PTH	20	Healthy adults	[211]
	Exercise	↓PTH	54	Chronic kidney disease patients	[212]
	Exercise	↑PTH	29	Boys and young men	[213]
	Exercise	↑PTH	11 (men)	Healthy adults	[214]
	Exercise	↑PTH	25	Healthy adults	[215]
	Exercise	↑PTH	12 (men)	Healthy adults	[216]
	Exercise	↔iPTH	100 (women)	Healthy adults	[217]
	Exercise	↑iPTH	21	Healthy adults	[218]
	Exercise	↑iPTH	7 (men)	Healthy adults	[219]
	Exercise	↓PTH	5 (women)	Healthy adults	[220]
	Exercise	↑iPTH	9 (men)	Healthy adults	[221]
	Exercise	↑PTH (during the exercise with the highest intensity)	10 (men)	Healthy adults	[222]
	Exercise	↑PTH (during the exercise) ↔PTH (postexercise period)	10 (men)	Healthy adults	[223]
	Exercise	↑PTH	10 (women)	Healthy adults	[104]
	Exercise	↑PTH	51 (men)	Healthy adults	[224]
	Exercise	↓iPTH (moderate exercise) ↑iPTH (intensive exercise)	21 (women)	Healthy adults	[225]
	Exercise	↑PTH	14 (women)	Healthy adults	[226]
	Exercise	↓PTH (with the onset of exercise) ↑PTH (intensive exercise)	10 (men)	Healthy adults	[227]
	Exercise	↑PTH	17 (men)	Healthy adults	[228]
	Exercise	↑PTH	100 (men)	Healthy adults	[229]
	Exercise	↑PTH	9 (men)	Healthy adults	[111]
	Exercise	↑PTH	26 (women)	Healthy adults	[230]
	Exercise	↑PTH	18	Healthy adults	[112]
	Exercise	↑iPTH	8 (men)	Healthy adults	[231]
	Exercise	↔PTH	6 (men)	Healthy adults	[232]
	Exercise	↑PTH	6 (men)	Healthy adults	[109]
	Exercise	↑PTH	19 (men)	Healthy adults	[107]
	Exercise	↔PTH	13 (men)	Healthy adults	[110]
	Exercise	↑PTH	27 (men)	Healthy adults	[20]

BMI, body mass index; iPTH, intact parathyroid hormone; PTH, parathyroid hormone. Decreased (↓), unchanged (↔), increased (↑).

**Table 2:** Lifestyle factors that affect calcitonin levels in humans.

	Factor	Effect on Hormone Levels	Number of Participants	Participants	Reference
**Smoking**	Smoking	↔Calcitonin	294 (women)	Healthy adults	[18]
	Smoking	↑Calcitonin	9340	People with type 2 diabetes	[48]
	Smoking	↑Calcitonin	142 (men)	Healthy adults	[233]
	Smoking	↑Calcitonin	58	Healthy adults	[133]
	Smoking	↑Calcitonin	120 (men)	Healthy adults	[234]
	Smoking	↑Calcitonin	6341 (men)	Healthy adults	[17]
**Alcohol consumption**	Alcohol	↔Calcitonin	26	Healthy adults	[136]
	Alcohol	↔Calcitonin	93	Healthy adults	[96]
	Alcohol	↓Calcitonin (in a heavy drinking group)	47	Alcoholics	[95]
	Alcohol	↑Calcitonin	50	Alcoholics + controls	[94]
**Increased BMI**	↑BMI	↔Calcitonin	467	Patients with Hashimoto’s thyroiditis	[235]
	↑BMI	↓Calcitonin	294	Healthy adults	[18]
	↑BMI	↑Calcitonin	9340	People with type 2 diabetes	[48]
	↑BMI	↑Calcitonin	287	Healthy adults	[233]
	↑BMI	↔Calcitonin	4638	Healthy adults	[17]
	↑BMI	↑Calcitonin	31	Patients with chronic kidney disease on hemodialysis	[236]
**Vitamins and minerals**	Vitamin D supplementation	↔Calcitonin	270 (women)	Healthy adults	[75]
	Zinc infusion	↓Calcitonin	38	Patients of short stature, diabetes mellitus, and controls	[83]
	High dietary zinc	↓Calcitonin	21	Healthy adults	[86]
	High dietary copper	↔Calcitonin	21	Healthy adults	[86]
**Exercise**	Exercise	↔Calcitonin	9 (men)	Healthy adults	[111]
	Exercise	↔Calcitonin	18	Healthy adults	[112]
	Exercise	↔Calcitonin	6 (men)	Healthy adults	[109]
	Exercise	↑Calcitonin	19 (men)	Healthy adults	[107]
	Exercise	↔Calcitonin	13 (men)	Healthy adults	[110]
	Exercise	↔Calcitonin	27 (men)	Healthy adults	[20]
	Raloxifene combined with aerobic exercise	↑Calcitonin	70	Patients with osteoporosis	[108]

BMI, body mass index. Decreased (↓), unchanged (↔), increased (↑).

### 3.2. Pollutants

#### 3.2.1. Heavy Metals

Various heavy metals, such as cadmium (Cd), arsenic (As), and lead (Pb), affect PTH levels. Most studies have shown that PTH levels decrease after cadmium exposure (Table 3). Schutte et al., explained the decrease in PTH levels after cadmium exposure as a consequence of the direct osteotoxic effect of cadmium [18]. Exposure to cadmium leads to a decrease in bone density, resulting in increased release of calcium from bone tissue. The result of increased calcium release is the decrease in PTH levels [18]. In addition, cadmium has been shown to have a toxic effect on parathyroid glands [237]. However, some studies did not observe any effect [238,239,240] or observed an increase [241,242] in PTH levels in subjects exposed to cadmium. Studies in experimental animals observed an increase in PTH levels after cadmium exposure [243]. Arsenic exposure did not affect PTH levels [244]. Most studies reported an increase in PTH levels in subjects exposed to lead (Table 3). Lead inhibits 1α-hydroxylase (the enzyme responsible for the production of 1,25(OH)2D) [245], and since PTH and 25(OH)D are in an inverse relationship, a decrease in 25(OH)D levels results in an increase in PTH levels. PTH levels were also measured in Gulf War I veterans who were exposed to uranium, and it was shown that uranium exposure led to a decrease in PTH levels [246].

We found only one study that analyzed the influence of heavy metals on calcitonin levels. Schutte et al., observed an increase in calcitonin levels after cadmium exposure [18]. A study in rats showed that exposure to cadmium and lead decreased calcitonin levels [243,247]. Exposure of laying hens to cadmium led to a decrease in calcitonin levels [248], while a study in goldfish found no changes in calcitonin levels after cadmium exposure (although exposure to methylmercury increased calcitonin levels) [249].

#### 3.2.2. Chemicals

Only a few studies have investigated the effect of chemicals on PTH levels in humans (Table 3). Exposure to persistent organochlorine compounds (p,p′-diphenyldichloroethene (p,p′-DDE) and polychlorinated biphenyls (PCBs)) did not affect PTH levels [132,250]. Exposure to perfluoroalkyl substances (PFAS) led to an increase in PTH levels [251]. Di Nisio et al. suggested that perfluoro-octanoic acid (PFOA) binds to vitamin D receptors, causing reduced 1,25(OH)D activity, which in turn increases PTH levels [251]. Fluoride exposure increases PTH levels [252]. According to researchers, excess fluoride alters calcium metabolism and potentially leads to secondary hyperparathyroidism (reviewed in [253]). Exposure to perchlorate, thiocyanate, and nitrate has led to a decrease in PTH levels, but the underlying mechanism of this action is not yet clear [19].

Data on the effect of chemicals and pesticides on calcitonin levels in humans are scarce. A study on goldfish has shown that bisphenol A inhibits the release of calcitonin [249]. Aroclor 1254 (PCB) increased calcitonin expression in rat thyroid [254]. Because many chemicals have an endocrine disruptive effect [255], further studies are needed on the impact of chemicals and pesticides on PTH and calcitonin levels.

**Table 3:** Pollutants affecting PTH and calcitonin levels in humans.

	Factor	Effect on Hormone Levels	Number of Participants	Participants	Reference
**Heavy metals**	Arsenic	↔PTH–	196	Healthy adults	[256]
	Arsenic	↔iPTH	774	Children and new-borns	[244]
	Cadmium	↓PTH	719 (women)	Healthy adults	[34]
	Cadmium	↓PTH	85 (women)	Healthy adults	[257]
	Cadmium	↓PTH	51 (men)	Participants exposed to cadmium	[258]
	Cadmium	↔PTH	46	Participants exposed to cadmium for a long period (some suffering from decreased tubular function)	[240]
	Cadmium	↔PTH	41 (women)	Subjects with renal tubular dysfunction caused by exposure to cadmium	[259]
	Cadmium	↓iPTH	306	Chronic peritoneal dialysis patients	[260]
	Cadmium in urine (maternal)	↓PTH (in boys) ↑PTH (in girls)	504	504 children in a mother–child cohort	[242]
	Cadmium in erythrocytes (maternal)	↑PTH (in boys) ↓PTH (in girls)	504		
	Cadmium	↔PTH	60	Patients with renal tubular damage caused by exposure to cadmium and healthy controls	[238]
	Cadmium	↑PTH	53	Patients with renal tubular damage caused by exposure to cadmium and healthy controls	[241]
	Cadmium	↓PTH (association lost after adjustment for smoking)	908 (women)	Healthy adults	[132]
	Cadmium	↓PTH, ↑Calcitonin	294 (women)	Healthy adults	[18]
	Cadmium	↔PTH	146	Healthy adults	[239]
	Lead	↑PTH	89	Healthy adults	[245]
	Lead	↔PTH	719 (women)	Healthy adults	[34]
	Lead	↔PTH	51	Dialysis patients	[261]
	Lead	↑PTH	146 (men)	Healthy adults	[262]
	Lead	↑iPTH	315	Chronic peritoneal dialysis patients	[263]
	Lead	↑PTH	115	Hemodialysis patients	[264]
	Lead	↔PTH	47	Healthy adults	[265]
	Lead	↑PTH	73 (women)	Healthy adults	[266]
	Lead	↑iPTH	93	Hemodialysis patients	[267]
	Uranium	↔iPTH	35	Gulf War I veterans exposed to uranium	[268]
	Uranium	↓iPTH	35	Gulf War I veterans exposed to uranium	[246]
**Chemicals**	Persistent organochlorine compounds (CB-153)	↔PTH	908 (women)	Healthy adults	[132]
	Persistent organochlorine compounds (p,p’-DDE)	↔PTH			
	PFAS	↑PTH	100 (men)	Healthy adults	[251]
	PCBs (exposed prenatally)	↔PTH	110	Children in a mother–child cohort	[250]
	Fluoride	↑PTH	196	Healthy adults	[256]
	Fluoride	↑PTH	84	Patients with endemic fluorosis and healthy controls	[252]
	Fluoride	↓PTH (in pregnant women)	180	Pregnant women and their new-borns	[269]
		↔PTH (in new-borns)			
	Lithium	↔iPTH	178	Mother–child cohort	[270]
	Perchlorate	↓PTH	2207 (women)	Healthy adults	[19]
	Nitrate	↓PTH	4265	Healthy adults	[19]
	Thiocyanate	↓PTH	4265	Healthy adults	[19]

iPTH, intact parathyroid hormone; PCB, polychlorinated biphenyl; PFAS, perfluoroalkyl substances; p,p′-DDE, p,p′-diphenyldichloroethene; PTH, parathyroid hormone. Decreased (↓), unchanged (↔), increased (↑).

## 4. Conclusions

In this review, we gave an insight into environmental factors that affect the levels of PTH and calcitonin, two hormones that regulate calcium and phosphate homeostasis. We included literature discussing lifestyle factors (smoking, BMI, diet, alcohol, and exercise) and pollutants (heavy metals and chemicals) (Figure 1). In terms of lifestyle factors, most studies have shown a decrease in PTH levels in smokers, a positive correlation between BMI and PTH, an increase in PTH levels during exercise, and a decrease in PTH levels after vitamin D and calcium intake (Table 1). The results of studies on the impact of alcohol consumption and intake of different types of food and micronutrients (except for vitamin D and calcium) showed great variability (Table 1). Regarding studies that analyzed the effect of pollutants on PTH levels, the clearest relationship was between PTH and cadmium, with PTH levels decreasing after cadmium exposure (Table 3). While arsenic exposure did not affect PTH levels, lead exposure resulted in increased PTH levels (Table 3). Several studies have investigated the influence of chemicals on PTH levels in humans. Moreover, data on the effect of chemicals and heavy metals on calcitonin levels in humans are scarce, and most of the knowledge, to date, relies on studies in experimental animals. As for the relationship between lifestyle factors and calcitonin, several studies have been conducted on humans and have given great variability in results. The most consistent results were related to smoking (an increase in calcitonin levels was observed in smokers) (Table 2). Given the important role that PTH and calcitonin play in maintaining calcium and phosphate homeostasis in the body, additional studies on the influence of environmental and genetic factors that could affect the levels of these two hormones are extremely important.

## Data Availability

Not applicable.

## Abbreviations

1,25(OH)2D3, 1α,25-dihydroxyvitamin D; 25(OH)D, 25-hydroxyvitamin D; As, arsenic; BMD, bone mineral density; BMI, body mass index; Ca, calcium; Cd, cadmium; F, fluoride; FGF-23, fibroblast growth factor 23; iPTH, intact parathyroid hormone; Mg, magnesium; Pb, lead; PCB, polychlorinated biphenyl; PFAS, perfluoroalkyl substances; PFOA, perfluoro-octanoic acid; p,p-DDE, p,p-diphenyldichloroethene; PTH, parathyroid hormone; Zn, zinc.
